# Association between Cabrol shunt and new-onset atrial fibrillation after acute type A aortic dissection surgery: a retrospective study

**DOI:** 10.3389/fcvm.2026.1859883

**Published:** 2026-06-15

**Authors:** Hao Song, Shiying Gao, Zhihao Yang, Chao Fu, Zhongxu Huang, Chunxiao Liu

**Affiliations:** 1 Department of Cardiovascular Surgery, Qilu Hospital of Shandong University, Jinan, China; 2 Institute of Thoracoscopy in Cardiac Surgery, Shandong University, Jinan, China; 3 School of Nursing, Shandong Xiehe University, Jinan, China

**Keywords:** acute type A aortic dissection, Cabrol shunt, new-onset postoperative atrial fibrillation, propensity score matching, risk factors

## Abstract

**Objective:**

To examine the association between Cabrol shunt and the incidence of new-onset postoperative atrial fibrillation (POAF) after surgery for acute type A aortic dissection (AAAD).

**Methods:**

A total of 240 patients who underwent AAAD surgery were retrospectively enrolled, including 185 patients treated with a Cabrol shunt and 55 patients without a Cabrol shunt. Propensity score matching (PSM) was used to reduce treatment selection bias. Multivariable logistic regression was performed to identify factors associated with new-onset POAF.

**Results:**

Patients in the non-Cabrol shunt group had a higher observed incidence of new-onset POAF than those in the Cabrol shunt group (*P* = 0.013). This finding remained consistent across multiple sensitivity analyses as well as the number needed to treat analysis. Multivariable logistic regression showed that age and aortic cross-clamp time were independent factors influencing the occurrence of new-onset POAF in patients with AAAD.

**Conclusions:**

Cabrol shunt use was associated with a lower observed incidence of new-onset POAF in patients undergoing AAAD surgery. Aortic cross-clamp time may be related to this association. Age was also independently associated with new-onset POAF.

## Introduction

Acute type A aortic dissection (AAAD) poses a substantial cardiovascular risk, including severe complications such as hypotension, shock, periaortic hematoma, mesenteric ischemia, and cerebral injury (1). In recent years, with the increasing number of AAAD surgeries and optimization of surgical and assistive techniques, the postoperative morbidity and mortality rates of AAAD patients have significantly decreased (2). Despite meticulous surgical techniques and the routine employment of hemostatic agents, postoperative oozing from suture lines remains a common issue following open repair of AAAD (3). This oozing is primarily due to the suture line's location within the affected aortic segment, reduced blood coagulability following dissection, and the prolonged duration of cardiopulmonary bypass (4). While in most cases the oozing is eventually controlled, achieving complete hemostasis can take a considerable amount of time, and some severe oozing may require a second operation to stop the bleeding. The Cabrol shunt is a surgical technique employed to manage uncontrolled hemorrhage during the graft replacement of the ascending aorta and aortic root within the aneurysmal sac (inclusion technique) (5). Currently, the Cabrol shunt is utilized at some Chinese clinical centers for the prophylactic, routine, or remedial management of surgical repairs for AAAD (6).

Atrial fibrillation (AF), a prevalent arrhythmia, often develops subsequent to cardiac surgery (7). According to the literature, the incidence of postoperative atrial fibrillation (POAF) following AAAD surgery ranges from 19.6% to 57.2% (8). Despite technological advances, the incidence of POAF, one of the most common complications after AAAD surgery, has not significantly improved (9). Although previous studies have identified risk factors for new-onset POAF following AAAD surgery, the association between the implementation of Cabrol shunt and the incidence of new-onset POAF in AAAD patients remains poorly understood. This study examined perioperative outcomes in patients with AAAD and evaluated the association between Cabrol shunt use and the incidence of new-onset POAF.

## Methods

### Patient selection and outcome definition

**Figure 1:**
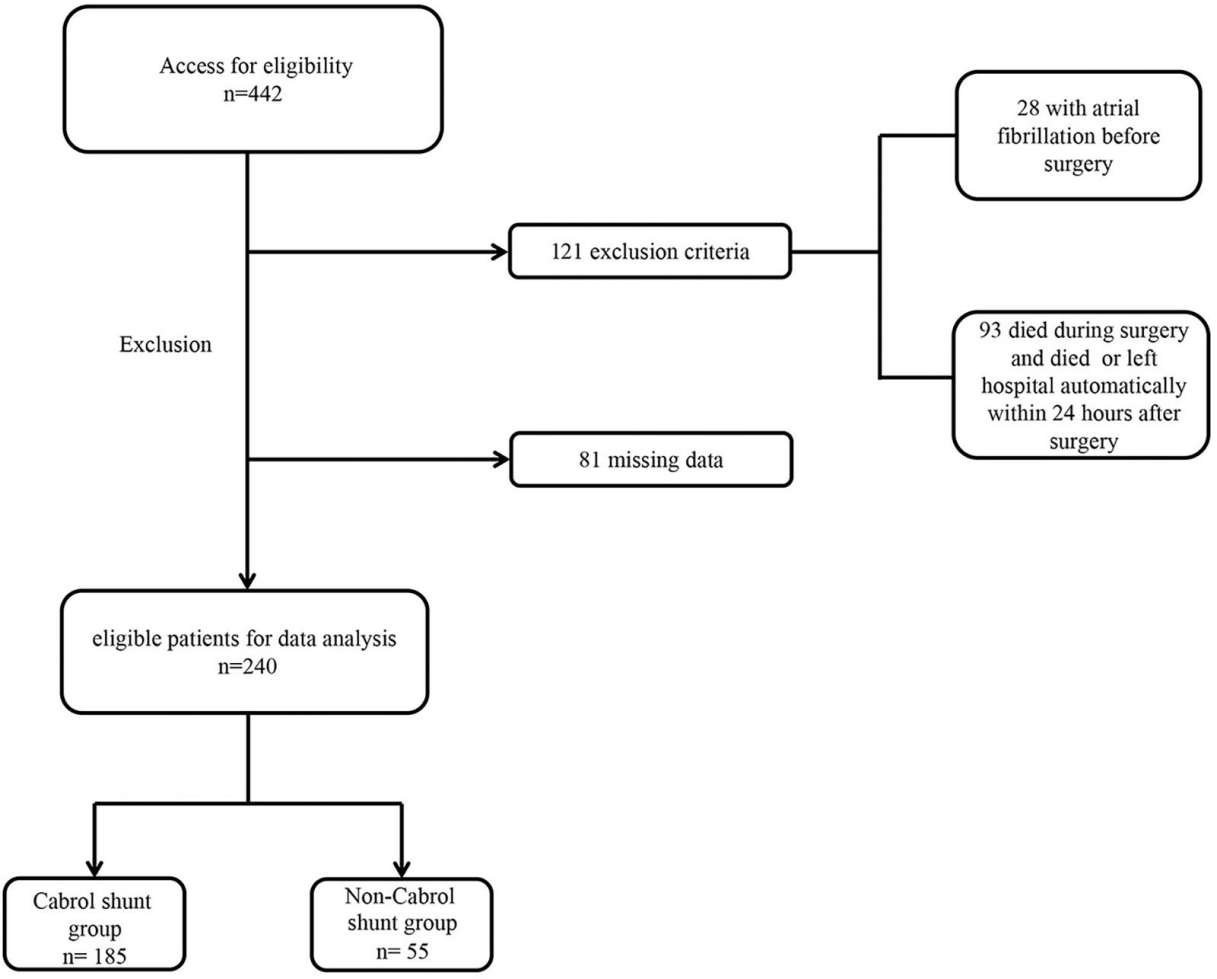
Flow chart for selection of the study population.

This retrospective observational study was reported in accordance with the Strengthening the Reporting of Observational Studies in Epidemiology (STROBE) statement (10). A retrospective review of patients diagnosed with AAAD who underwent surgery at Qilu Hospital, Shandong University, was conducted between December 2017 and December 2022. Inclusion Criteria: (1) Confirmed diagnosis of AAAD via CT angiography of the aorta. (2) Absence of a prior history of AF attacks. (3) AAAD patients scheduled for surgery within 24 h of diagnosis. Exclusion Criteria: (1) Patients younger than 18 years of age. (2) Patients exhibiting temporary or persistent AF, atrial flutter, or other supraventricular tachycardia prior to surgery. (3) Death occurring within 24 h post-operative. (4) Uncontrolled exacerbation of the primary disease within 24 h post-operatively, prompting family requests for voluntary discharge or withdrawal of treatment. (5) Substantial loss of clinical data. Ultimately, 240 patients were included in the analysis cohort. The study cohort was divided into two groups based on the application of the Cabrol shunt: the Cabrol shunt group and the non-Cabrol shunt group (Figure 1). Patients with missing data in variables required for the planned multivariable and propensity score analyses were excluded using complete-case analysis. The majority of missing data were related to echocardiographic parameters. In many of these cases, echocardiography had been performed at outside hospitals before transfer, and repeat echocardiographic examination was not routinely performed after admission because patients underwent emergency surgery shortly after arrival at our center. To assess the potential selection bias introduced by missing-data exclusion, available baseline and clinical characteristics were compared between patients included in the final analytic cohort and those excluded because of missing data.

The primary endpoint was new-onset POAF, characterized by atrial fibrillation lasting five minutes or more after surgery, according to the Society of Thoracic Surgeons criteria (11). New-onset POAF was identified based on continuous ECG monitoring, routine 12-lead ECGs, and medical record documentation during the postoperative hospitalization. All patients received continuous ECG monitoring during ICU stay. After ICU discharge, rhythm surveillance was continued according to standard ward practice, including routine 12-lead ECG examination and additional ECG assessment whenever symptoms or abnormal rhythm findings were suspected. The same postoperative monitoring strategy was applied to both the Cabrol shunt and non-Cabrol shunt groups, and no group-specific rhythm monitoring protocol was used. Rhythm-related management, including beta-blocker or amiodarone use and electrolyte correction, was administered according to clinical indications and institutional perioperative practice rather than Cabrol shunt status. During perioperative intensive care, electrolytes were routinely monitored by arterial blood gas analysis three times daily, and abnormalities were corrected according to institutional protocols.

### Cabrol shunt definition and surgical strategy

In the present study, the term “Cabrol shunt” refers specifically to a perigraft-to-right atrial shunt used for the management of perigraft bleeding during AAAD repair, rather than to the modified Cabrol coronary reconstruction technique. The same general Cabrol shunt strategy was used in all included cases: residual perigraft bleeding around the reconstructed ascending aorta or aortic root was redirected into the right atrium through a surgically created perigraft-to-right atrial communication. Cabrol shunt construction was not randomly assigned or applied according to a mandatory standardized protocol. Instead, the decision was made at the discretion of the operating surgeon according to intraoperative findings, bleeding control requirements, and the perceived risk of persistent perigraft or suture-line bleeding. No formal change in the institutional indication for Cabrol shunt construction was documented during the study period. Nevertheless, because the decision was made intraoperatively at the discretion of the operating surgeon, subtle changes in surgeon preference or practice over time cannot be completely excluded. Therefore, Cabrol shunts could be constructed either as a planned prophylactic strategy in selected AAAD patients considered to be at high risk of difficult bleeding control, or as a rescue/remedial maneuver when uncontrolled perigraft or suture-line bleeding was encountered intraoperatively. Preset Cabrol shunt was not used as a standardized strategy specifically intended to reduce cardiopulmonary bypass time. All Cabrol shunts in this cohort were constructed using non-valved vascular grafts, with no use of valved shunts or valved conduits such as Contegra grafts. Concomitant procedures were performed when clinically indicated and were recorded in the present study. In particular, some patients underwent concomitant aortic root procedures, including Bentall or David procedures. Because of the limited number of such cases, these procedures were not listed separately in Table 1, but were summarized as “Root replacement”. Patients who underwent concomitant coronary artery bypass grafting were not included in this study, because coronary revascularization may influence postoperative atrial fibrillation and introduce potential confounding.

**Table 1:** Baseline characteristics.

Characteristics	Total (*n* = 240)	Cabrol shunt group (*n* = 185)	Non-Cabrol shunt group (*n* = 55)	*P* value
Demographics				
Gender, female (%)	73 (30.42%)	49 (26.49%)	24 (43.64%)	0.024
Age (years)	51.00 (42.00, 61.00)	51.00 (43.00, 59.00)	54.00 (41.00, 65.00)	0.391
Weight (kg)	74.00 (65.00, 83.75)	75.00 (65.00, 85.00)	70.00 (60.00, 80.00)	0.058
Clinical history and risk factors				
Smoking history (%)	91 (37.92%)	72 (38.92%)	19 (34.55%)	0.557
Drinking history (%)	115 (47.92%)	95 (51.35%)	20 (36.36%)	0.051
Hypertension (%)	164 (68.33%)	124 (67.03%)	40 (72.73%)	0.425
Diabetes (%)	11 (4.58%)	9 (4.86%)	2 (3.64%)	0.702
preACS (%)	27 (11.25%)	17 (9.19%)	10 (18.18%)	0.064
COPD (%)	15 (6.25%)	12 (6.49%)	3 (5.45%)	0.781
Laboratory profiles				
WBC (×10^9^/L)	9.86 (7.66,12.28)	9.89 (7.65, 12.36)	9.79 (7.86, 12.07)	0.632
RBC (×10^12^/L)	4.01 (3.63,4.40)	4.02 (3.69, 4.42)	3.92 (3.36, 4.32)	0.076
HGB (g/L)	123.50 (110.25, 135.00)	124.00 (112.00, 135.00)	121.00 (102.50, 135.00)	0.138
PLT(×10^9^/L)	167.50 (136.00, 209.75)	167.00 (137.00, 206.00)	170.00 (135.00, 211.50)	0.907
PTINR	1.11 (1.05，1.20)	1.12 (1.06, 1.20)	1.09 (1.04, 1.17)	0.444
APTT (s)	30.65 (28.43，33.78)	30.20 (28.50, 33.70)	31.30 (28.40, 34.25)	0.463
FIB (g/L)	3.28 (2.52，4.31)	3.26 (2.46, 4.31)	3.33 (2.76, 3.96)	0.650
CKMB (ng/mL)	1.80 (0.80,6.30)	1.70 (0.70, 6.20)	2.00 (1.00, 6.30)	0.406
CRP (mg/L)	4.75 (3.40, 5.05)	4.20 (3.30, 5.00)	4.90 (3.45, 5.05)	0.612
NT-proBNP (pg/mL)	798 (440, 1,629)	776 (437, 1,660)	829 (488, 1,532)	0.586
Echocardiogram profiles				
LA (mm)	36.31 ± 6.47	35.97 ± 6.58	37.45 ± 6.02	0.122
LV (mm)	45.00 (42.00, 50.00)	45.00 (42.00, 50.00)	47.00 (42.00, 51.50)	0.287
RA (mm)	44.29 ± 7.17	44.12 ± 6.75	44.84 ± 8.47	0.569
RV (mm)	23.00 (20.00, 25.00)	23.00 (20.00, 24.00)	23.00 (21.00, 25.00)	0.152
LVEF	0.61 (0.58, 0.65)	0.60 (0.58, 0.65)	0.63 (0.59, 0.65)	0.273
Procedure characteristics				
Root replacement (%)	92 (38.33%)	76 (41.08%)	16 (29.09%)	0.108
Surgery time (min)	477.69 ± 93.51	480.81 ± 95.81	467.18 ± 85.30	0.315
Cardiopulmonary bypass time (min)	216.00 (188.00, 250.00)	219.00 (189.00, 253.00)	206.00 (189.00, 238.50)	0.324
Aortic cross-clamp time (min)	137.00 (117.00, 159.00)	134.00 (112.00, 156.00)	146.00 (132.00, 162.50)	0.002
Circulatory arrest time (min)	26.00 (19.00, 35.00)	22.00 (18.00, 29.00)	39.00 (36.00, 42.00)	<0.001
Perioperative outcomes				
New-onset POAF (%)	96 (40.00%)	66 (35.68%)	30 (54.55%)	0.012
Operative mortality (%)	18 (7.50%)	14 (7.57%)	4 (7.27%)	0.942

LA, left atrial diameter; LV, Left ventricular diameter; preACS, previous acute coronary syndrome; POAF, postoperative atrial fibrillation; RA, right atrial diameter; RV, right ventricular diameter.

Root replacement included Bentall procedure and valve-sparing aortic root replacement David procedure.

### Statistical analysis

Statistical analysis was performed using R software and SPSS statistical software. Data are presented as mean ± SD for normally distributed data and as median (P25, P75) for non-normally distributed data. Comparisons between groups were made using the independent t-test for normally distributed data and the rank-sum test for non-normally distributed data. Categorical variables were reported as counts and percentages, with differences between groups assessed using the *χ*² test.

First, univariable logistic regression was used to evaluate the crude association between Cabrol shunt use and new-onset POAF. Second, multivariable logistic regression was performed to adjust for potential confounders. Third, propensity score matching was performed as a sensitivity analysis to further reduce baseline imbalance between the Cabrol shunt and non-Cabrol shunt groups (12). The propensity score was estimated using logistic regression models with Cabrol shunt use as the dependent variable. Three propensity score models were constructed using different covariate sets. Model 1 included demographic variables, including gender, age, and weight, as well as clinical history and risk factors, including smoking history, drinking history, hypertension, diabetes, preoperative acute coronary syndrome(preACS), and chronic obstructive pulmonary disease. Model 2 included laboratory profiles, including WBC, NEU, RBC, LYM, HGB, PLT, PT-INR, APTT, FIB, CK-MB, CRP, and NT-proBNP, as well as echocardiographic profiles, including LA, LV, RA, RV, and LVEF. Model 3 included all covariate domains, including demographic variables, clinical history and risk factors, laboratory profiles, and echocardiographic profiles.

In each propensity score model, patients were matched using 2:1 nearest-neighbor matching without replacement, with a caliper width of 0.2 standard deviations of the logit of the propensity score. Common support was assessed by inspecting the overlap of propensity score distributions between the Cabrol shunt and non-Cabrol shunt groups, and patients outside the region of common support were not retained in the matched cohort. Patients with missing data for variables included in the corresponding propensity score model were handled using complete-case analysis. Covariate balance before and after matching was assessed using standardized mean differences rather than *P* values, with an absolute SMD <0.10 considered indicative of acceptable balance (13). The association between Cabrol shunt use and new-onset POAF in the matched cohort was evaluated using conditional logistic regression to account for the matched structure.After PSM, the measured baseline characteristics were generally comparable between the Cabrol shunt and non-Cabrol shunt groups, as shown in Supplementary Tables S1, S2. Covariate balance before and after matching was further assessed using standardized mean differences, which are provided in Supplementary Tables S3, S4. Subgroup analyses were conducted to further explore interactions between the Cabrol shunt procedure and the occurrence of new-onset POAF. For all analyses, statistical significance was set at *P* < 0.05. We further performed an exploratory absolute risk difference analysis and calculated the NNT-style estimate as a descriptive measure (14). This estimate was not intended to imply a causal treatment effect.

Patients in the Cabrol shunt and non-Cabrol shunt groups were further stratified according to the occurrence of new-onset POAF. Univariable logistic regression was performed to evaluate crude associations between candidate variables and POAF. Exploratory multivariable logistic regression was then conducted using a limited set of clinically relevant variables selected based on clinical plausibility, prior evidence, and univariable findings, while avoiding overfitting relative to the number of POAF events. Given the limited sample size, particularly in the non-Cabrol shunt group, subgroup regression analyses were interpreted as exploratory and parsimonious models were used. The linearity assumption in the logit for continuous variables, including age and aortic cross-clamp time, was assessed using the Box-Tidwell approach before inclusion in the logistic regression models.

## Results

### Patient characteristics and outcome

A total of 240 patients were included in the analysis, with a median age of 51 years (IQR, 42–61) and a gender distribution of 73 females. Of these, 185 patients underwent the Cabrol shunt procedure.And the incidence of new-onset POAF was 35.68% in the Cabrol shunt group and 54.55% in the non-Cabrol shunt group(*P* = 0.012). Operative mortality was 7.57% in the Cabrol shunt group and 7.27% in the non-Cabrol shunt group, with no statistically significant difference between the two groups (*P* = 0.942). Patient characteristics and perioperative outcomes, stratified by the presence or absence of the Cabrol shunt procedure, are shown in Table 1. Among the screened AAAD patients, 81 were excluded because of missing data. The missing data were mainly related to echocardiographic parameters, largely because some patients had undergone echocardiography at outside hospitals before transfer and did not undergo repeat echocardiographic examination after admission because of emergency surgery. As shown in Supplementary Table S5, most available baseline and clinical characteristics were comparable between patients included in the final analytic cohort and those excluded because of missing data. However, NT-proBNP differed between the included and excluded patients. Therefore, although the excluded patients were broadly similar to the included patients based on available variables, potential selection bias related to complete-case analysis cannot be completely excluded.

### Association of Cabrol shunt with new-onset POAF

As depicted in Table 2, the incidence of new-onset POAF was significantly higher in the non-Cabrol shunt group compared to the Cabrol shunt group (OR 2.16, 95% CI 1.18–4.01; *P* = 0.013). After adjusting for factors such as gender, age, weight, clinical history and risk factors, as well as laboratory profiles and echocardiogram findings, the risk remained similar in multivariable analysis (OR 2.17, 95% CI 1.02–4.66; *p* = 0.047) and propensity score matching analysis (OR 2.29, 95% CI 1.16–4.51; *p* = 0.017). After propensity score matching, the measured baseline characteristics were generally comparable between the Cabrol shunt and non-Cabrol shunt groups in the matched cohort, as shown in Supplementary Table S1. Covariate balance was further assessed using standardized mean differences, and the corresponding SMD results are provided in Supplementary Table S3. And after adjustment, Cabrol shunt use was not significantly associated with operative mortality.

**Table 2:** Associations between Cabrol shunt and new-onset POAF in the crude analysis, multivariable analysis and propensity score matching analysis.

Analysis	New-onset POAF	*P* value
No. of events/No. Of patients at risk (%)		
Cabrol shunt	66/185 (35.68%)	
Non-Cabrol shunt	30/55 (54.55%)	
Crude analysis—OR (95%CI)	2.16 (1.18–4.01)	0.013
Multivariable analysis^a^	2.25 (1.14–4.47)	0.019
Propensity score matching analysis^a^	2.57 (1.33–5.04)	0.005
Multivariable analysis^b^	2.21 (1.08–4.54)	0.030
Propensity score matching analysis^b^	2.18 (1.10–4.32)	0.026
Multivariable analysis^c^	2.17 (1.02–4.66)	0.047
Propensity score matching analysis^c^	2.29 (1.16–4.51)	0.017

a Adjustment for gender, age, weight, clinical history and risk factors(Smoking history, Drinking history, Hypertension, Diabetes, preACS, COPD).

b Adjustment for laboratory profiles (WBC, RBC, HGB, PLT, PTINR, APTT, FIB, CKMB, CRP, NT-proBNP) and Echocardiogram profiles (LA, LV, RA, RV, LVEF).

c Adjustment for gender, age, weight, clinical history and risk factors, as well as laboratory profiles and Echocardiogram profiles.

The Cabrol shunt group was used as the reference group.

### Subgroup analysis of root procedure

**Figure 2:**
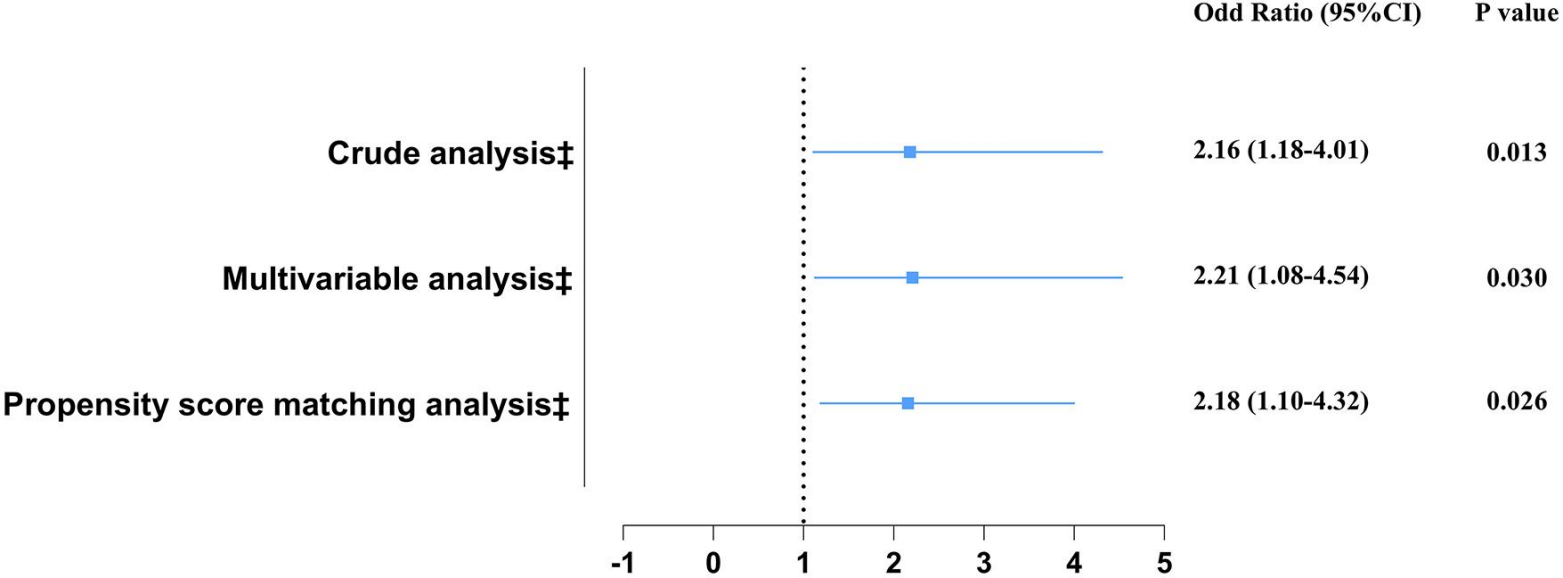
Subgroup analysis of association between cabrol shunt and new-onset POAF. ‡Adjustment for gender, age, weight, clinical history and risk factors, as well as laboratory profiles and Echocardiogram profiles.

A subgroup analysis was performed on patients who did not receive root replacement surgery to explore potential interactions between the use of Cabrol shunts and the occurrence of new-onset POAF. The rate of non-replacement was 58.91% (109/185) in the Cabrol shunt group and 70.91% (39/55) in the non-Cabrol shunt group. Among patients without root replacement, the incidence of new-onset POAF was higher in the non-Cabrol shunt group compared to the Cabrol shunt group, (OR 2.16, 95% CI 1.18–4.01; *p* = 0.013). This association remained significant after additional adjustment for multivariable analysis (OR 2.21, 95% CI 1.08–4.54; *p* = 0.030) and propensity score matching analysis (OR 2.18, 95% CI 1.10–4.32; *p* = 0.026) (Figure 2). In the subgroup without root replacement, the measured baseline characteristics were generally comparable between the Cabrol shunt and non-Cabrol shunt groups after propensity score matching, as shown in Supplementary Table S2. The corresponding standardized mean differences before and after matching are provided in Supplementary Table S4.

### Exploratory absolute risk difference analysis

Overall, the observed absolute risk difference corresponded to an exploratory NNT-style estimate of 5 patients (95% CI 3–15), corresponding to one fewer observed case of new-onset POAF in the Cabrol shunt group compared with the non-Cabrol shunt group. In the subgroup without root replacement, the corresponding exploratory NNT-style estimate was 4 patients (95% CI 2–8)**.** These estimates were descriptive and should not be interpreted as evidence that Cabrol shunt use causally prevents new-onset POAF.

### Associated risk factors of new-onset POAF

The overall incidence of new-onset POAF was 35.68% (66/185) in the Cabrol shunt group and 54.55% (30/55) in the non-Cabrol shunt group. Additionally, patient characteristics and perioperative outcomes, stratified by the presence or absence of new-onset POAF in both the Cabrol shunt and non-Cabrol shunt groups, are shown in Supplementary Table S6.

In the Cabrol shunt group, age (OR, 1.036; 95% CI, 1.009–1.064; *P* = 0.009) and aortic cross-clamp time (OR, 1.009; 95% CI, 1.002–1.019; *P* = 0.044) were independently associated with new-onset POAF in exploratory multivariable logistic regression analysis. The Box-Tidwell test showed no significant violation of the linearity assumption for age (*P* = 0.372) or aortic cross-clamp time (*P* = 0.246). In the non-Cabrol shunt group, only age was independently associated with new-onset POAF (OR, 1.041; 95% CI, 1.003–1.081; *P* = 0.035). The Box-Tidwell test showed no significant violation of the linearity assumption for age in the non-Cabrol shunt group (*P* = 0.289) (Table 3).

**Table 3:** Multivariable logistic regression analysis showing the risk variables of new-onset POAF in Cabrol shunt group and Non-Cabrol shunt group.

Cabrol shunt group			Non-Cabrol shunt group		
Variable	Odds ratio (95% CI)	*P* value	Variable	Odds ratio (95% CI)	*P* value
Age	1.036 (1.009–1.064)	0.009	Age	1.041 (1.003–1.081)	0.035
Drinking history	1.880 (0.963–3.669)	0.064			
preACS	2.751 (0.960–7.885)	0.060			
RA	1.051 (0.999–1.105)	0.053			
Aortic cross-clamp time	1.009 (1.002–1.019)	0.044			

## Discussion

In our study, the incidence of new-onset POAF among patients with AAAD who underwent surgery was 40.0% (96/240), aligning with previously reported findings (8). It is believed that most new-onset POAF is self-limiting and converts to sinus rhythm before hospital discharge (15). In our research, we found that in the majority of patients, new-onset POAF was transient and typically resolved within one week of surgery. However, 6.25% (6/96) of patients still had symptoms of AF one week after surgery, and all of these patients were in the group that did not receive a Cabrol shunt. In this study, we observed a higher incidence of new-onset POAF in patients who did not receive a Cabrol shunt than in those who did. A similar association was observed in the subgroup of patients who underwent surgery without root replacement. Similar results were observed in the number needed to treat analysis. In the present cohort, Cabrol shunt use was not significantly associated with operative mortality. This finding is consistent with the China 5A study, which reported that Cabrol shunt was not associated with either a substantially lower or higher risk of operative mortality, regardless of aortic root replacement (16). Therefore, the observed association between Cabrol shunt use and new-onset POAF should be interpreted separately from mortality outcomes and should not be taken to imply a survival benefit.

In the current study, aortic cross-clamp time was longer in patients who did not receive a Cabrol shunt, and this variable was associated with new-onset POAF in the Cabrol shunt subgroup. Previous studies have also reported an association between prolonged aortic cross-clamp time and a higher incidence of POAF (8). A longer aortic cross-clamp time will result in a longer period of myocardial ischemia. Kota RK et al. demonstrated that myocardial ischemic time represents the critical factor influencing the incidence of POAF (17). In addition, prolonged cardioplegic arrest may require a greater cumulative dose of hyperkalemic cardioplegic solution, which has been proposed as a potential contributor to postoperative arrhythmogenesis (18). Although this mechanism has not been conclusively demonstrated by previous clinical and experimental studies, its potential causative role should not be dismissed. Consequently, myocardial ischemia and the elevated potassium infusion necessary to maintain prolonged cardioplegic arrest may be pivotal contributors to the emergence of new-onset POAF. Importantly, aortic cross-clamp time may have different conceptual roles in the association between Cabrol shunt use and new-onset POAF. It may act as a mediator if Cabrol shunt construction contributes to shorter operative ischemic time; alternatively, it may function as a confounder or simply as a marker of greater operative complexity. These possibilities cannot be disentangled in the present retrospective observational design. Therefore, aortic cross-clamp time should not be regarded as an established mechanism underlying the observed association between Cabrol shunt use and POAF. Rather, it should be interpreted as an exploratory factor that may be related to the observed association.

The Cabrol shunt is constructed by connecting the aneurysm wrap to the right atrial appendage to recirculate the bleeding back into the circulation (19). This procedure can transform a bleeding site into a dry, clear surgical field, effectively controlling bleeding during the surgical repair of AAAD. Numerous studies have corroborated that patients who received a Cabrol shunt for AAAD had less post-operative exudation than those who did not (4). Patients experiencing high postoperative 24-hour leakage may be more susceptible to developing new-onset POAF. A growing body of evidence indicates that massive oozing within the pericardial space may contribute to a highly prooxidant and proinflammatory environment, which can potentially trigger new-onset POAF through breakdown products, activation of the coagulation cascade, and oxidative burst (20). Several clinical studies indirectly support this hypothesis, reporting that the incidence of POAF can be reduced by surgical drainage of the pericardium during the postoperative period (21).

Despite the presence of a Cabrol shunt following the AAAD procedure, the study showed that the channel closed rapidly and likely had minimal impact on the atrial structure (5). AF following aortic arch repair with deep hypothermic circulatory arrest is a prevalent phenomenon, with reported incidences reaching as high as 52.7% (22). The non-Cabrol shunt group has been observed to exhibit a longer duration of deep hypothermic arrest, which may potentially increase the risk of new-onset POAF in these patients.

The study results indicated that age independently affected the development of new-onset POAF in patients with AAAD, regardless of whether they received a Cabrol shunt or not (*P* < 0.05). Elderly patients typically exhibited a heightened prevalence of chronic diseases, alongside degenerative alterations in cardiovascular structure and electrophysiological abnormalities. Clinical epidemiological studies have consistently demonstrated an increasing risk of AF as age advances (23). However, no significant age difference was observed between patients treated with the Cabrol shunt and those who did not receive the Cabrol shunt.

In the Cabrol shunt group, aortic cross-clamp time was also associated with new-onset POAF. Recent studies have also reported that longer aortic cross-clamp time is associated with an increased risk of POAF after cardiac surgery (24). The potential mechanism may involve atrial ischemia resulting from inadequate myocardial protection during cardiac arrest, as well as hyperthermia and oxidative stress induced by myocardial ischemia and reperfusion injury (25). In the current study, patients in the non-Cabrol shunt group had longer aortic cross-clamping times compared to those in the Cabrol shunt group. This could be a significant factor contributing to the higher incidence of new-onset POAF in the patients who did not receive a Cabrol shunt. However, the limited sample size of the non-Cabrol group may restrict the ability to accurately reflect the relationship between aortic cross-clamp time and the incidence of new-onset POAF. Future research could expand the sample size to validate this hypothesis.

### Limitations

This study has several limitations. First, as a retrospective, single-centre, non-randomized observational study, selection bias and confounding by indication could not be completely eliminated. Cabrol shunt construction was determined by the operating surgeon according to intraoperative findings and bleeding control requirements rather than by random assignment. Although multivariable adjustment, propensity score matching, and sensitivity analyses were performed, residual confounding from unmeasured factors related to disease anatomy, operative complexity, surgeon preference, surgical era, and perioperative management may still have influenced the observed association. Therefore, the findings should be interpreted as hypothesis-generating observational associations rather than evidence of a causal protective effect of Cabrol shunt use against POAF.

Second, 81 patients were excluded because of missing data, mainly missing echocardiographic parameters. In many cases, echocardiography had been performed at outside hospitals before transfer, and repeat echocardiography was not routinely performed after admission because of emergency surgery. Although most available baseline and clinical characteristics were comparable between included and excluded patients, NT-proBNP differed between the two groups; therefore, selection bias related to complete-case analysis cannot be completely excluded. In addition, serum magnesium was not routinely available, and residual confounding related to unmeasured electrolyte abnormalities may remain.

Third, although patients with known preoperative AF, atrial flutter, or other supraventricular tachycardias were excluded, undetected preoperative paroxysmal AF may still have been present because of the emergency nature of AAAD surgery. Although the same postoperative rhythm monitoring strategy was applied to both groups, POAF ascertainment after ICU discharge may have been limited by routine ward-based surveillance. Detailed patient-level data on rhythm-related management, including beta-blockers, amiodarone, magnesium supplementation, and electrolyte correction, were not uniformly available.

Fourth, the non-Cabrol shunt group was relatively small, which may limit the stability of subgroup regression estimates and the reliability of complex multivariable modelling. Postoperative stroke and ICU stay were not prespecified outcomes and were not consistently captured with predefined definitions; therefore, they were not included in the current analysis.

Finally, the present study has limited ability to infer mechanisms. In particular, the causal role of aortic cross-clamp time could not be determined because it may represent a mediator, confounder, or marker of operative complexity. Postoperative shunt closure was also not systematically assessed using a standardized imaging protocol; therefore, shunt patency, spontaneous closure, and potential long-term hemodynamic complications related to persistent shunting, such as right heart failure or pulmonary hypertension, require further evaluation in future prospective studies.

## Conclusion

The use of a Cabrol shunt was associated with a lower incidence of new-onset postoperative atrial fibrillation after surgery for acute type A aortic dissection. This association may be partly related to shorter aortic cross-clamp time. Furthermore, age appears to be a significant determinant affecting the incidence of new-onset POAF in patients with AAAD. Further prospective studies are needed to confirm these findings.

## Data Availability

The original contributions presented in the study are included in the article/Supplementary Material, further inquiries can be directed to the corresponding author.

## References

[1] Li S Wei D Wang Z Song H Cheng S Zhao X. Effect on surgery outcomes owing to the interval between onset of symptoms and surgery of patients with acute type A aortic dissection. Emerg Crit Care Med. (2022) 2(2):67–72. 10.1097/EC9.0000000000000032

[2] Elsayed RS Cohen RG Fleischman F Bowdish ME. Acute type A aortic dissection. Cardiol Clin. (2017) 35(3):331–45. 10.1016/j.ccl.2017.03.004 28683905

[3] Toole JM Stroud MR Ikonomidis JS. Salvage periaortic pericardial baffle equalizes mortality in bleeding patients undergoing aortic surgery. J Thorac Cardiovasc Surg. (2014) 148(1):151–5. 10.1016/j.jtcvs.2013.08.008 24113020

[4] Zhang H Wu X Fang G Qiu Z Chen LW. Is it justified to apply a modified Cabrol fistula in surgical repair of acute type A aortic dissection? J Thorac Cardiovasc Surg. (2019) 158(5):1307–14.e2. 10.1016/j.jtcvs.2018.12.082 30737112

[5] Raghuram A Kalyanasundaram A Babcock M Zafar MA Ziganshin B Elefteriades J. Long-term follow-up of Cabrol fistula for uncontrollable bleeding: a life-saving procedure. JTCVS Tech. (2023) 21:1–6. 10.1016/j.xjtc.2023.06.010 37854823 PMC10580030

[6] Cai Z Yan T Zhang B Su C Zhang H Zhang W. Analysis of the effect of preset Cabrol in total arch replacement for type A aortic dissection. J Thorac Dis. (2025) 17(8):5454–65. 10.21037/jtd-2025-349 40950922 PMC12433102

[7] Yang Z Liu C Fu C Zhao X. A nomogram for individualized prediction of new-onset postoperative atrial fibrillation in acute type A aortic dissection patients: a retrospective study. Front Cardiovasc Med. (2024) 11:1429680. 10.3389/fcvm.2024.1429680 39234610 PMC11371795

[8] Zhao R Wang Z Cao F Song J Fan S Qiu J. New-onset postoperative atrial fibrillation after total arch repair is associated with increased in-hospital mortality. J Am Heart Assoc. (2021) 10(18):e021980. 10.1161/JAHA.121.021980 34533045 PMC8649499

[9] Qureshi M Ahmed A Massie V Marshall E Harky A. Determinants of atrial fibrillation after cardiac surgery. Rev Cardiovasc Med. (2021) 22(2):329–41. 10.31083/j.rcm2202040 34258901

[10] von Elm E Altman DG Egger M Pocock SJ Gøtzsche PC Vandenbroucke JP. The strengthening the reporting of observational studies in epidemiology (STROBE) statement: guidelines for reporting observational studies. Ann Intern Med. (2007) 147(8):573–7. 10.7326/0003-4819-147-8-200710160-00010 17938396

[11] Arakawa M Miyata H Uchida N Motomura N Katayama A Tamura K. Postoperative atrial fibrillation after thoracic aortic surgery. Ann Thorac Surg. (2015) 99(1):103–8. 10.1016/j.athoracsur.2014.08.019 25440282

[12] Austin PC. An Introduction to propensity score methods for reducing the effects of confounding in observational studies. Multivariate Behav Res. (2011) 46(3):399–424. 10.1080/00273171.2011.568786 21818162 PMC3144483

[13] Austin PC. Balance diagnostics for comparing the distribution of baseline covariates between treatment groups in propensity-score matched samples. Stat Med. (2009) 28(25):3083–107. 10.1002/sim.3697 19757444 PMC3472075

[14] Suter K Briel M Günther J. The number needed to treat (NNT) and the number needed to harm (NNH). Med Monatsschr Pharm. (2015) 38(3):103–6. 26364396

[15] Gudbjartsson T Helgadottir S Sigurdsson MI Taha A Jeppsson A Christensen TD. New-onset postoperative atrial fibrillation after heart surgery. Acta Anaesthesiol Scand. (2020) 64(2):145–55. 10.1111/aas.13507 31724159

[16] Liu H Sun BQ Qian SC Sun MY Shao YF Ding Y. Contemporary use and outcome of cabrol shunt in type A aortic dissection surgery: insight from China 5A study. Open Heart. (2023) 10(2):e002465. 10.1136/openhrt-2023-002465 38070883 PMC10729034

[17] Kota RK Gemelli M Dimagli A Suleiman SS Moscarelli M Dong T. Patterns of cytokine release and association with new onset of post-cardiac surgery atrial fibrillation. Front Surg. (2023) 10:1205396. 10.3389/fsurg.2023.1205396 37325422 PMC10266410

[18] Caretta Q Mercanti CA De Nardo D Chiarotti F Scibilia G Reale A. Ventricular conduction defects and atrial fibrillation after coronary artery bypass grafting. Multivariate analysis of preoperative, intraoperative and postoperative variables. Eur Heart J. (1991) 12(10):1107–11. 10.1093/oxfordjournals.eurheartj.a059845 1782937

[19] Ma M, Mohamed MA, Li Y, Wei X. A modified wrapping-internal shunt method for hemostasis in bentall procedure. *Med Arch*. (2016) 70(4):321–3. 10.5455/medarh.2016.70.321-323 PMC5034998 27703300

[20] St-Onge S Perrault LP Demers P Boyle EM Gillinov AM Cox J. Pericardial blood as a trigger for postoperative atrial fibrillation after cardiac surgery. Ann Thorac Surg. (2018) 105(1):321–8. 10.1016/j.athoracsur.2017.07.045 29174782

[21] Gaudino M Sanna T Ballman KV Robinson NB Hameed I Audisio K. Posterior left pericardiotomy for the prevention of atrial fibrillation after cardiac surgery: an adaptive, single-centre, single-blind, randomised, controlled trial. Lancet. (2021) 398(10316):2075–83. 10.1016/S0140-6736(21)02490-9 34788640

[22] Suero OR Ali-Ahmed F Lapin B Mlodynia AR McCarthy PM. Postoperative atrial fibrillation (POAF) after cardiac surgery: clinical practice review. J Thorac Dis. (2024) 16(1):815–33. 10.21037/jtd-23-1616 PMC10944787 38505057

[23] Guo Y Tian Y Wang H Si Q Wang Y Lip GYH. Prevalence, incidence, and lifetime risk of atrial fibrillation in China: new insights into the global burden of atrial fibrillation. Chest. (2015) 147(1):109–19. 10.1378/chest.14-0321 24921459

[24] Taha A Särnblad S Jeppsson A Hjärpe A Martinsson A Nielsen SJ. Cardiopulmonary bypass management and risk of new-onset postoperative atrial fibrillation in cardiac surgery. Interdiscip Cardiovasc Thorac Surg. (2023) 37(3):ivad153. 10.1093/icvts/ivad153 37713475 PMC10533753

[25] Gaudino M Di Franco A Rong LQ Piccini JP Mack MJ. Postoperative atrial fibrillation: from mechanisms to treatment. Eur Heart J. (2023) 44(12):1027–39. 10.1093/eurheartj/ehad019 PMC10226752 36721960

