# Supplementary material for: Association between Cabrol shunt and new-onset atrial fibrillation after acute type A aortic dissection surgery: a retrospective study
Source: Front Cardiovasc Med. 2026 Jun 15;13:1859883. doi: 10.3389/fcvm.2026.1859883 (PMC13310719; doi:10.3389/fcvm.2026.1859883)
Supplement: Supplementary file 1 [file Table1.doc]

**Supplementary Table S1.** Adjusted patient characteristics, operative data and outcomes.

|  | Total (165) | Cabrol shunt group (110) | Non-Cabrol shunt group (55) | P value |
| --- | --- | --- | --- | --- |
| **Demographic profiles** |  |  |  |  |
| Female gender (%) | 65 (39.4%) | 41 (37.3%) | 24 (43.6%) | 0.563 |
| Age (years) | 52.00 (44.00, 60.00) | 51.00 (45.00, 59.00) | 54.00 (41.00, 65.00) | 0.440 |
| Weight (kg) | 70.00 (62.00, 80.00) | 70.00 (63.00, 80.00) | 70.00 (60.00, 80.00) | 0.829 |
| **Clinical history and risk factors** |  |  |  |  |
| Smoking history (%) | 60 (36.4%) | 41 (37.3%) | 19 (34.5%) | 0.864 |
| Drinking history (%) | 63 (38.2%) | 43 (39.1%) | 20 (36.4%) | 0.856 |
| Hypertension (%) | 120 (72.7%) | 124 (72.7%) | 40 (72.7%) | 1.000 |
| Diabetes (%) | 9 (5.5%) | 7 (6.4%) | 2 (3.6%) | 0.716 |
| preACS (%) | 24 (14.5%) | 14 (12.7%) | 10 (18.2%) | 0.482 |
| COPD (%) | 10 (6.1%) | 7 (6.4%) | 3 (5.5%) | 1.000 |
| **Laboratory profiles** |  |  |  |  |
| WBC (×10^9^ /L) | 9.63 (7.54,12.18) | 9.48 (7.35, 12.35) | 9.79 (7.86, 12.07) | 1.000 |
| RBC (×10^12^ /L) | 3.95 (3.56,4.37) | 3.96 (3.62, 4.39) | 3.92 (3.36, 4.32) | 0.271 |
| HGB (g/L) | 122.00 (109.00, 135.00) | 122.00 (110.00, 135.00) | 121.00 (102.50, 135.00) | 0.446 |
| PLT(×10^9^ /L) | 161.00 (134.00, 206.00) | 159.50 (133.25, 199.25) | 170.00 (135.00, 211.50) | 0.560 |
| PTINR | 1.11 (1.06，1.20) | 1.13 (1.06, 1.20) | 1.09 (1.04, 1.17) | 0.292 |
| APTT (s) | 30.80 (28.50，34.20) | 30.80 (28.55, 34.10) | 31.30 (28.40, 34.25) | 0.765 |
| FIB (g/L) | 3.20 (2.43，4.31) | 3.09 (2.41, 4.31) | 3.33 (2.76, 3.96) | 0.485 |
| CKMB (ng/ml) | 1.70 (0.80,6.30) | 1.50 (0.70, 5.88) | 2.00 (1.00, 6.30) | 0.206 |
| CRP (mg/L) | 4.77 (3.42, 5.09) | 4.78 (3.39, 5.10) | 4.85 (3.45, 5.05) | 0.793 |
| NT-proBNP (pg/mL) | 808 (458, 1582) | 804 (452, 1602) | 820 (490, 1529) | 0.802 |
| **Echocardiogram profiles** |  |  |  |  |
| LA (mm) | 35.83 ± 6.08 | 35.18 ± 5.98 | 37.45 ± 6.02 | 0.015 |
| LV (mm) | 45.00 (41.00, 50.00) | 45.00 (40.00, 48.75) | 47.00 (42.00, 51.50) | 0.053 |
| RA (mm) | 43.60 ± 7.24 | 42.98 ± 6.49 | 44.84 ± 8.47 | 0.121 |
| RV (mm) | 22.00 (20.00, 24.00) | 22.00 (20.00, 24.00) | 23.00 (21.00, 25.00) | 0.033 |
| LVEF | 0.61 (0.58, 0.65) | 0.60 (0.58, 0.65) | 0.63 (0.59, 0.65) | 0.296 |
| **Procedure characteristics** |  |  |  |  |
| Root replacement (%) | 55(33.3%) | 39 (35.5%) | 16 (29.1%) | 0.521 |
| Surgerytime (min) | 470.20 ± 91.64 | 471.71 ± 94.99 | 467.18 ± 85.30 | 0.766 |
| Cardiopulmonary bypass time (min) | 214.00 (182.00, 245.00) | 215.00 (182.00, 250.00) | 206.00 (189.00, 238.50) | 0.676 |
| Aortic cross-clamp time (min) | 136.00 (117.00, 157.00) | 131.00 (112.00, 154.00) | 146.00 (132.00, 162.50) | 0.002 |
| Circulatory arrest time (min) | 27.00 (21.00, 37.00) | 23.00 (19.00, 29.00) | 39.00 (36.00, 42.00) | <0.001 |
| **Perioperative outcomes** |  |  |  |  |
| New-onset POAF (%) | 65(39.4%) | 35 (31.8%) | 30 (54.5%) | 0.008 |
| Operative mortality (%) | 14(8.48%) | 9 (8.18%) | 5(9.09%) | 0.771 |

*Adjustment for gender, age, weight , clinical history and risk factors( hypertension, DM, preACS, COPD, smoking, drinking).

|  | Total (165) | Cabrol shunt group (110) | Non-Cabrol shunt group (55) | P value |
| --- | --- | --- | --- | --- |
| **Demographic profiles** |  |  |  |  |
| Female gender (%) | 59 (35.8%) | 35 (31.8%) | 24 (43.6%) | 0.180 |
| Age (years) | 52.00 (45.00, 63.00) | 51.50 (45.00, 62.75) | 54.00 (41.00, 65.00) | 0.691 |
| Weight (kg) | 70.00 (64.00, 80.00) | 72.00 (65.00, 81.00) | 70.00 (60.00, 80.00) | 0.286 |
| **Clinical history and risk factors** |  |  |  |  |
| Smoking history (%) | 55 (33.3%) | 36 (32.7%) | 19 (34.5%) | 0.999 |
| Drinking history (%) | 68 (41.2%) | 48 (43.6%) | 20 (36.4%) | 0.480 |
| Hypertension (%) | 112 (67.9%) | 72 (65.5%) | 40 (72.7%) | 0.406 |
| Diabetes (%) | 6 (3.6%) | 4 (3.6%) | 2 (3.6%) | 1.000 |
| preACS (%) | 25 (15.2%) | 15 (13.6%) | 10 (18.2%) | 0.486 |
| COPD (%) | 9 (5.5%) | 6 (5.5%) | 3 (5.5%) | 1.000 |
| **Laboratory profiles** |  |  |  |  |
| WBC (×10^9^ /L) | 9.50 (7.52, 12.10) | 9.30 (7.30, 12.00) | 9.79 (7.86, 12.07) | 0.742 |
| RBC (×10^12^ /L) | 3.89 (3.51, 4.30) | 3.87 (3.55, 4.27) | 3.92 (3.36, 4.32) | 0.874 |
| HGB (g/L) | 120.00 (108.00, 131.00) | 119.50 (109.00, 128.50) | 121.00 (102.50, 135.00) | 0.739 |
| PLT(×10^9^ /L) | 174.00 (138.00, 216.00) | 176.00 (139.00, 218.00) | 170.00 (135.00, 211.50) | 0.493 |
| PTINR | 1.10 (1.04, 1.17) | 1.10 (1.04, 1.16) | 1.09 (1.04, 1.17) | 0.942 |
| APTT (s) | 30.70 (28.10, 34.30) | 30.20 (27.95, 34.20) | 31.30 (28.40, 34.25) | 0.464 |
| FIB (g/L) | 3.36 (2.66, 4.28) | 3.37 (2.62, 4.34) | 3.33 (2.76, 3.96) | 0.917 |
| CKMB (ng/ml) | 1.70 (0.80, 4.90) | 1.60 (0.70, 4.10) | 2.00 (1.00, 6.30) | 0.097 |
| CRP (mg/L) | 4.79 (3.38, 5.09) | 4.72 (3.34, 5.10) | 4.90 (3.45, 5.05) | 0.756 |
| NT-proBNP (pg/mL) | 811 (457, 1593) | 801 (443, 1618) | 829 (488, 1532) | 0.793 |
| **Echocardiogram profiles** |  |  |  |  |
| LA (mm) | 36.54 ± 6.42 | 36.10 ± 6.55 | 37.45 ± 6.02 | 0.210 |
| LV (mm) | 45.00 (42.00, 51.00) | 45.00 (42.00, 50.00) | 47.00 (42.00, 51.50) | 0.447 |
| RA (mm) | 44.76 ± 7.21 | 44.72 ± 6.53 | 44.84 ± 8.47 | 0.921 |
| RV (mm) | 23.00 (21.00, 25.00) | 23.00 (21.00, 25.00) | 23.00 (21.00, 25.00) | 0.884 |
| LVEF | 0.61 (0.59, 0.65) | 0.60 (0.58, 0.65) | 0.63 (0.59, 0.65) | 0.682 |
| **Procedure characteristics** |  |  |  |  |
| Root replacement (%) | 59 (35.8%) | 43 (39.1%) | 16 (29.1%) | 0.273 |
| Surgerytime (min) | 469.13 ± 93.55 | 470.10 ± 97.40 | 467.18 ± 85.30 | 0.844 |
| Cardiopulmonary bypass time (min) | 212.00 (184.00, 244.00) | 213.00 (183.50, 246.00) | 206.00 (189.00, 238.50) | 0.683 |
| Aortic cross-clamp time (min) | 137.00 (120.00, 158.00) | 133.00 (116.00, 154.00) | 146.00 (132.00, 162.50) | 0.003 |
| Circulatory arrest time (min) | 30.00 (20.00, 38.00) | 24.00 (19.00, 31.00) | 39.00 (36.00, 42.00) | <0.001 |
| **Perioperative outcomes** |  |  |  |  |
| New-onset POAF (%) | 70 (42.4%) | 40 (36.4%) | 30 (54.5%) | 0.035 |
| Operative mortality (%) | 13（7.88%） | 8（7.27%） | 5（9.09%） | 0.762 |

†Adjustment for laboratory profiles (WBC, NEU, RBC, LYM, HGB, PLT, PTINR, APTT, FIB, CKMBCRP, NT-proBNP) and Echocardiogram profiles ( LA, LV, RA, RV, LVEF).

|  | Total (165) | Cabrol shunt group (110) | Non-Cabrol shunt group (55) | P value |
| --- | --- | --- | --- | --- |
| **Demographic profiles** |  |  |  |  |
| Female gender (%) | 65 (39.4%) | 41 (37.3%) | 24 (43.6%) | 0.536 |
| Age (years) | 52.00 (44.00, 62.00) | 51.50 (45.00, 62.00) | 54.00 (41.00, 65.00) | 0.681 |
| Weight (kg) | 71.00 (63.00, 80.00) | 71.00 (64.00, 80.25) | 70.00 (60.00, 80.00) | 0.459 |
| **Clinical history and risk factors** |  |  |  |  |
| Smoking history (%) | 59 (35.8%) | 40 (36.4%) | 19 (34.5%) | 0.954 |
| Drinking history (%) | 64 (38.8%) | 44 (40.0%) | 20 (36.4%) | 0.778 |
| Hypertension (%) | 110 (66.7%) | 70 (63.6%) | 40 (72.7%) | 0.321 |
| Diabetes (%) | 9 (5.5%) | 7 (6.4%) | 2 (3.6%) | 0.716 |
| preACS (%) | 24 (14.5%) | 14 (12.7%) | 10 (18.2%) | 0.482 |
| COPD (%) | 9 (5.5%) | 6 (5.5%) | 3 (5.5%) | 1.000 |
| **Laboratory profiles** |  |  |  |  |
| WBC (×10^9^ /L) | 9.60 (7.41, 12.02) | 9.48 (7.22, 12.02) | 9.79 (7.86, 12.07) | 0.704 |
| RBC (×10^12^ /L) | 3.94 (3.55, 4.31) | 3.95 (3.60, 4.30) | 3.92 (3.36, 4.32) | 0.776 |
| HGB (g/L) | 122.00 (109.00, 134.00) | 121.50 (111.00, 132.50) | 121.00 (102.50, 135.00) | 0.674 |
| PLT(×10^9^ /L) | 171.00 (139.00, 207.00) | 171.50 (141.00, 205.50) | 170.00 (135.00, 211.50) | 0.914 |
| PTINR | 1.10 (1.04, 1.17) | 1.10 (1.04, 1.17) | 1.09 (1.04, 1.17) | 0.944 |
| APTT (s) | 30.70 (28.30, 34.10) | 30.50 (28.20, 34.00) | 31.30 (28.40, 34.25) | 0.673 |
| FIB (g/L) | 3.25 (2.59, 4.14) | 3.21 (2.51, 4.23) | 3.33 (2.76, 3.96) | 0.608 |
| CKMB (ng/ml) | 1.65 (0.80, 5.20) | 1.50 (0.70, 4.05) | 2.00 (1.00, 6.30) | 0.142 |
| CRP (mg/L) | 4.77 (3.39, 5.08) | 4.71 (3.34, 5.10) | 4.90 (3.45, 5.05) | 0.716 |
| NT-proBNP (pg/mL) | 815 (457, 1587) | 804 (444, 1615) | 829 (488, 1532) | 0.760 |
| **Echocardiogram profiles** |  |  |  |  |
| LA (mm) | 36.58 ± 6.08 | 36.15 ± 6.10 | 37.45 ± 6.02 | 0.198 |
| LV (mm) | 46.00 (43.00, 51.00) | 45.50 (43.00, 50.50) | 47.00 (42.00, 51.50) | 0.725 |
| RA (mm) | 45.00 ± 7.03 | 45.08 ± 6.25 | 44.84 ± 8.47 | 0.837 |
| RV (mm) | 23.00 (21.00, 25.00) | 23.00 (21.00, 25.00) | 23.00 (21.00, 25.00) | 0.991 |
| LVEF | 0.61 (0.59, 0.65) | 0.60 (0.58, 0.65) | 0.63 (0.59, 0.65) | 0.589 |
| **Procedure characteristics** |  |  |  |  |
| Root replacement (%) | 59 (35.8%) | 43 (39.1%) | 16 (29.1%) | 0.275 |
| Surgerytime (min) | 464.80 ± 92.90 | 463.60 ± 96.90 | 467.18 ± 85.30 | 0.811 |
| Cardiopulmonary bypass time (min) | 212.00 (183.00, 244.00) | 214.00 (178.00, 246.00) | 206.00 (189.00, 238.50) | 0.929 |
| Aortic cross-clamp time (min) | 135.00 (118.00, 156.00) | 128.00 (113.00, 153.00) | 146.00 (132.00, 162.50) | <0.001 |
| Circulatory arrest time (min) | 27.00 (20.00, 37.00) | 22.00 (18.00, 28.00) | 39.00 (36.00, 42.00) | <0.001 |
| **Perioperative outcomes** |  |  |  |  |
| New-onset POAF (%) | 68 (41.2%) | 38 (34.5%) | 30 (54.5%) | 0.022 |
| Operative mortality (%) | 14(8.48%) | 10(9.09%) | 4(7.27%) | 0.776 |

‡Adjustment for gender, age, weight, clinical history and risk factors, as well as laboratory profiles and Echocardiogram profiles.
