# Supplementary material for: Association between Cabrol shunt and new-onset atrial fibrillation after acute type A aortic dissection surgery: a retrospective study
Source: Front Cardiovasc Med. 2026 Jun 15;13:1859883. doi: 10.3389/fcvm.2026.1859883 (PMC13310719; doi:10.3389/fcvm.2026.1859883)
Supplement: Supplementary file 2 [file Table2.doc]

**Supplementary Table S2.** Adjusted patient characteristics, operative data and outcomes (No root replacement surgery ).

|  | Total (117) | Cabrol shunt group (78) | Non-Cabrol shunt group (39) | P value |
| --- | --- | --- | --- | --- |
| **Demographic profiles** |  |  |  |  |
| Female gender (%) | 41 (35.0%) | 25 (32.1%) | 16 (41.0%) | 0.451 |
| Age (years) | 55.00 (47.00, 63.00) | 54.00 (48.00, 61.00) | 57.00 (43.00, 67.00) | 0.410 |
| Weight (kg) | 74.00 (64.00, 82.00) | 75.00 (65.00, 83.00) | 70.00 (60.00, 80.00) | 0.259 |
| **Clinical history and risk factors** |  |  |  |  |
| Smoking history (%) | 43 (36.8%) | 31 (39.7%) | 12 (30.8%) | 0.456 |
| Drinking history (%) | 47 (40.2%) | 33 (42.3%) | 14 (35.9%) | 0.641 |
| Hypertension (%) | 85 (72.6%) | 56 (71.8%) | 29 (74.4%) | 0.942 |
| Diabetes (%) | 4 (3.4%) | 3 (3.8%) | 1 (2.6%) | 1.000 |
| preACS (%) | 17 (14.5%) | 10 (12.8%) | 7 (17.9%) | 0.643 |
| COPD (%) | 8 (6.8%) | 6 (7.7%) | 2 (5.1%) | 0.897 |
| **Laboratory profiles** |  |  |  |  |
| WBC (×10^9^ /L) | 9.90 (7.60, 12.05) | 10.20 (7.80, 12.00) | 9.42 (7.25, 11.25) | 0.286 |
| RBC (×10^12^ /L) | 3.94 (3.56, 4.28) | 3.96 (3.63, 4.29) | 3.83 (3.34, 4.31) | 0.166 |
| HGB (g/L) | 121.00 (108.00, 134.00) | 122.00 (112.00, 134.00) | 120.00 (98.00, 135.00) | 0.291 |
| PLT(×10^9^ /L) | 157.00 (126.00, 198.00) | 160.00 (128.00, 192.00) | 150.00 (123.00, 202.00) | 0.836 |
| PTINR | 1.11 (1.04, 1.20) | 1.12 (1.03, 1.19) | 1.10 (1.06, 1.21) | 0.823 |
| APTT (s) | 30.80 (28.50, 34.20) | 30.20 (28.30, 34.00) | 31.60 (29.10, 35.40) | 0.196 |
| FIB (g/L) | 3.28 (2.50, 4.43) | 3.22 (2.42, 4.62) | 3.50 (2.75, 4.18) | 0.661 |
| CKMB (ng/ml) | 1.60 (0.80, 5.00) | 1.50 (0.70, 4.00) | 2.00 (1.00, 6.10) | 0.142 |
| CRP (mg/L) | 4.79 (3.38, 5.08) | 4.72 (3.34, 5.12) | 4.90 (3.45, 5.05) | 0.748 |
| NT-proBNP (pg/mL) | 812 (455, 1587) | 803 (445, 1608) | 829 (488, 1532) | 0.781 |
| **Echocardiogram profiles** |  |  |  |  |
| LA (mm) | 36.60 ± 6.07 | 36.18 ± 6.11 | 37.42 ± 5.98 | 0.235 |
| LV (mm) | 45.00 (43.00, 50.00) | 45.00 (43.00, 49.00) | 46.00 (42.50, 51.00) | 0.269 |
| RA (mm) | 44.98 ± 7.04 | 45.08 ± 6.33 | 44.78 ± 8.38 | 0.800 |
| RV (mm) | 23.00 (21.00, 25.00) | 23.00 (21.00, 25.00) | 24.00 (21.00, 25.00) | 0.508 |
| LVEF | 0.62 (0.60, 0.65) | 0.61 (0.60, 0.65) | 0.63 (0.60, 0.66) | 0.739 |
| **Procedure characteristics** |  |  |  |  |
| Surgerytime (min) | 466.10 ± 92.70 | 463.50 ± 96.80 | 471.30 ± 84.10 | 0.622 |
| Cardiopulmonary bypass time (min) | 199.00 (179.00, 226.00) | 202.00 (176.00, 230.00) | 194.00 (181.00, 220.00) | 0.751 |
| Aortic cross-clamp time (min) | 127.00 (109.00, 145.00) | 120.00 (103.00, 137.00) | 138.00 (127.00, 156.00) | <0.001 |
| Circulatory arrest time (min) | 27.00 (19.00, 37.00) | 22.00 (18.00, 28.00) | 39.00 (36.00, 42.00) | <0.001 |
| **Perioperative outcomes** |  |  |  |  |
| New-onset POAF (%) | 48 (41.0%) | 25 (32.1%) | 23 (59.0%) | 0.009 |
| Operative mortality (%) | 8 (6.84%) | 5 (6.41%) | 3 (7.69%) | 0.796 |

‡Adjustment for gender, age, weight, clinical history and risk factors, as well as laboratory profiles and Echocardiogram profiles.
