# Supplementary material for: Association between Cabrol shunt and new-onset atrial fibrillation after acute type A aortic dissection surgery: a retrospective study
Source: Front Cardiovasc Med. 2026 Jun 15;13:1859883. doi: 10.3389/fcvm.2026.1859883 (PMC13310719; doi:10.3389/fcvm.2026.1859883)
Supplement: Supplementary file 3 [file Table3.doc]

**Supplementary Table S3.** Standardized mean differences before and after propensity score matching.

| Variable | Before PSM SMD | After PSM SMD |
| --- | --- | --- |
| Female gender | 0.365 | 0.055 |
| Age | 0.198 | 0.072 |
| Weight | 0.337 | 0.000 |
| Smoking history | 0.091 | 0.021 |
| Drinking history | 0.306 | 0.037 |
| Hypertension | 0.124 | 0.020 |
| Diabetes | 0.061 | 0.038 |
| preACS | 0.264 | 0.026 |
| COPD | 0.044 | 0.036 |
| WBC | 0.030 | 0.052 |
| RBC | 0.158 | 0.064 |
| HGB | 0.144 | 0.096 |
| PLT | 0.056 | 0.153 |
| PT-INR | 0.300 | 0.200 |
| APTT | 0.268 | 0.095 |
| FIB | 0.061 | 0.179 |
| CK-MB | 0.075 | 0.104 |
| CRP | 0.572 | 0.057 |
| NT-proBNP | 0.063 | 0.020 |
| LA | 0.235 | 0.127 |
| LV | 0.307 | 0.158 |
| RA | 0.094 | 0.124 |
| RV | 0.000 | 0.000 |
| LVEF | 0.621 | 0.207 |

*Adjustment for gender, age, weight , clinical history and risk factors( hypertension, DM, preACS, COPD, smoking, drinking).

| Variable | Before PSM SMD | After PSM SMD |
| --- | --- | --- |
| Female gender | 0.365 | 0.245 |
| Age | 0.198 | 0.160 |
| Weight | 0.337 | 0.149 |
| Smoking history | 0.091 | 0.038 |
| Drinking history | 0.306 | 0.147 |
| Hypertension | 0.124 | 0.156 |
| Diabetes | 0.061 | 0.000 |
| preACS | 0.264 | 0.126 |
| COPD | 0.044 | 0.000 |
| WBC | 0.030 | 0.048 |
| RBC | 0.158 | 0.052 |
| HGB | 0.144 | 0.061 |
| PLT | 0.056 | 0.064 |
| PT-INR | 0.300 | 0.058 |
| APTT | 0.268 | 0.066 |
| FIB | 0.061 | 0.041 |
| CK-MB | 0.075 | 0.055 |
| CRP | 0.572 | 0.049 |
| NT-proBNP | 0.063 | 0.035 |
| LA | 0.235 | 0.054 |
| LV | 0.307 | 0.062 |
| RA | 0.094 | 0.018 |
| RV | 0.000 | 0.000 |
| LVEF | 0.621 | 0.068 |

†Adjustment for laboratory profiles (WBC, RBC, HGB, PLT, PTINR, APTT, FIB, CKMB, CRP, NT-proBNP) and Echocardiogram profiles ( LA, LV, RA, RV, LVEF).

| Variable | Before PSM SMD | After PSM SMD |
| --- | --- | --- |
| Female gender | 0.365 | 0.048 |
| Age | 0.198 | 0.063 |
| Weight | 0.337 | 0.052 |
| Smoking history | 0.091 | 0.018 |
| Drinking history | 0.306 | 0.044 |
| Hypertension | 0.124 | 0.035 |
| Diabetes | 0.061 | 0.022 |
| preACS | 0.264 | 0.054 |
| COPD | 0.044 | 0.029 |
| WBC | 0.030 | 0.037 |
| RBC | 0.158 | 0.046 |
| HGB | 0.144 | 0.058 |
| PLT | 0.056 | 0.049 |
| PT-INR | 0.300 | 0.065 |
| APTT | 0.268 | 0.059 |
| FIB | 0.061 | 0.043 |
| CK-MB | 0.075 | 0.051 |
| CRP | 0.572 | 0.047 |
| NT-proBNP | 0.063 | 0.033 |
| LA | 0.235 | 0.055 |
| LV | 0.307 | 0.067 |
| RA | 0.094 | 0.025 |
| RV | 0.000 | 0.000 |
| LVEF | 0.621 | 0.074 |

‡Adjustment for gender, age, weight, clinical history and risk factors, as well as laboratory profiles and Echocardiogram profiles.
