# Supplementary material for: Association between Cabrol shunt and new-onset atrial fibrillation after acute type A aortic dissection surgery: a retrospective study
Source: Front Cardiovasc Med. 2026 Jun 15;13:1859883. doi: 10.3389/fcvm.2026.1859883 (PMC13310719; doi:10.3389/fcvm.2026.1859883)
Supplement: Supplementary file 4 [file Table4.doc]

**Supplementary Table S4.** Standardized mean differences before and after propensity score matching(No root replacement surgery ).

| Variable | Before PSM SMD | After PSM SMD |
| --- | --- | --- |
| Female gender | 0.420 | 0.061 |
| Age | 0.260 | 0.074 |
| Weight | 0.330 | 0.069 |
| Smoking history | 0.122 | 0.044 |
| Drinking history | 0.245 | 0.059 |
| Hypertension | 0.168 | 0.038 |
| Diabetes | 0.044 | 0.027 |
| preACS | 0.215 | 0.064 |
| COPD | 0.082 | 0.035 |
| WBC | 0.110 | 0.052 |
| RBC | 0.230 | 0.058 |
| HGB | 0.205 | 0.066 |
| PLT | 0.098 | 0.049 |
| PT-INR | 0.285 | 0.062 |
| APTT | 0.236 | 0.071 |
| FIB | 0.138 | 0.054 |
| CK-MB | 0.116 | 0.069 |
| CRP | 0.460 | 0.046 |
| NT-proBNP | 0.118 | 0.041 |
| LA | 0.281 | 0.057 |
| LV | 0.358 | 0.069 |
| RA | 0.112 | 0.032 |
| RV | 0.066 | 0.022 |
| LVEF | 0.510 | 0.075 |

‡Adjustment for gender, age, weight, clinical history and risk factors, as well as laboratory profiles and Echocardiogram profiles.
