# Supplementary material for: Association between Cabrol shunt and new-onset atrial fibrillation after acute type A aortic dissection surgery: a retrospective study
Source: Front Cardiovasc Med. 2026 Jun 15;13:1859883. doi: 10.3389/fcvm.2026.1859883 (PMC13310719; doi:10.3389/fcvm.2026.1859883)
Supplement: Supplementary file 5 [file Table5.doc]

**Supplemental Table S5.** Comparison of Included and Excluded Patients.

| **Variable** | **Included patients (n=240)** | **Excluded patients (n=81)** | **SMD** | **P value** |
| --- | --- | --- | --- | --- |
| Cabrol shunt use (%) | 185 (77.08%) | 62 (76.54%) | 0.013 | 0.921 |
| Female gender (%) | 73 (30.42%) | 25 (30.86%) | 0.010 | 0.940 |
| Age (years) | 51 (42, 61) | 52 (43, 62) | 0.071 | 0.581 |
| Weight (kg) | 74 (65, 83.75) | 73 (64, 83) | 0.071 | 0.580 |
| Smoking history (%) | 91 (37.92%) | 31 (38.27%) | 0.007 | 0.955 |
| Drinking history (%) | 115 (47.92%) | 38 (46.91%) | 0.020 | 0.876 |
| Hypertension (%) | 164 (68.33%) | 56 (69.14%) | 0.017 | 0.893 |
| Diabetes (%) | 11 (4.58%) | 5 (6.17%) | 0.071 | 0.561 |
| preACS (%) | 27 (11.25%) | 10 (12.35%) | 0.034 | 0.789 |
| COPD (%) | 15 (6.25%) | 6 (7.41%) | 0.046 | 0.716 |
| WBC ( ×10⁹/L) | 9.86 (7.66, 12.28) | 10.05 (7.80, 12.45) | 0.055 | 0.668 |
| RBC (×10¹²/L) | 4.01 (3.63, 4.40) | 3.98 (3.60, 4.38) | 0.052 | 0.686 |
| HGB (g/L) | 123.5 (110.25, 135) | 122 (109.5, 135) | 0.081 | 0.535 |
| PLT (×10⁹/L) | 167.5 (136, 209.75) | 170 (137, 215) | 0.044 | 0.734 |
| PT-INR | 1.11 (1.05, 1.20) | 1.12 (1.05, 1.22) | 0.084 | 0.526 |
| APTT (s) | 30.65 (28.43, 33.78) | 30.95 (28.70, 34.10) | 0.075 | 0.560 |
| FIB (g/L) | 3.28 (2.52, 4.31) | 3.38 (2.60, 4.45) | 0.074 | 0.568 |
| CK-MB (ng/mL) | 1.80 (0.80, 6.30) | 2.00 (0.90, 6.80) | 0.047 | 0.718 |
| CRP (mg/L) | 4.75 (3.40, 5.05) | 4.85 (3.48, 5.15) | 0.081 | 0.529 |
| NT-proBNP (pg/mL) | 798 (440, 1629) | 1120 (620, 2260) | 0.303 | 0.030 |
| Root replacement (%) | 92 (38.33%) | 30 (37.04%) | 0.027 | 0.835 |
| Surgery time (min) | 477.69 ± 93.51 | 486.00 ± 95.00 | 0.088 | 0.496 |
| Cardiopulmonary bypass time (min) | 216 (188, 250) | 219 (189, 255) | 0.063 | 0.629 |
| Aortic cross-clamp time (min) | 137 (117, 159) | 139 (118, 162) | 0.063 | 0.630 |
| Circulatory arrest time (min) | 26 (19, 35) | 27 (20, 37) | 0.082 | 0.532 |
| New-onset POAF (%) | 96 (40.00%) | 34 (41.98%) | 0.040 | 0.754 |
| Operative mortality (%) | 18 (7.50%) | 7 (8.64%) | 0.042 | 0.740 |
