# Supplementary material for: Association between Cabrol shunt and new-onset atrial fibrillation after acute type A aortic dissection surgery: a retrospective study
Source: Front Cardiovasc Med. 2026 Jun 15;13:1859883. doi: 10.3389/fcvm.2026.1859883 (PMC13310719; doi:10.3389/fcvm.2026.1859883)
Supplement: Supplementary file 6 [file Table6.doc]

**Supplemental Table S6.** Baseline characteristic of the AAAD patients in Cabrol shunt group and Non-Cabrol shunt group.

| Cabrol shunt group (n=185) | | | | |
| --- | --- | --- | --- | --- |
|  | Total(185) | Non-POAF group (119) | POAF group (66) | P value |
| **Demographic profiles** |  |  |  |  |
| Female gender (%) | 49 (26.5%) | 32 (26.9%) | 17 (25.8%) | 0.867 |
| Age (years) | 51.00 (43.00, 59.00) | 49.00 (41.50,58.00) | 55.00 (48.00,63.00) | 0.010 |
| Weight (kg) | 75.00 (65.00, 85.00) | 75.00 (65.00, 85.00) | 74.50 (65.00, 85.00) | 0.595 |
| **Clinical history and risk factors** |  |  |  |  |
| Smoking history (%) | 72 (38.9%) | 44 (37.0%) | 28 (42.4%) | 0.466 |
| Drinking history (%) | 95 (51.4%) | 53 (44.5%) | 42 (63.6%) | 0.013 |
| Hypertension (%) | 124 (67.0%) | 76 (63.9%) | 48 (72.7%) | 0.219 |
| Diabetes (%) | 9 (4.9%) | 5 (4.2%) | 4 (6.1%) | 0.573 |
| preACS (%) | 17 (9.2%) | 7 (5.9%) | 10 (15.2%) | 0.037 |
| COPD (%) | 12 (6.5%) | 8 (6.7%) | 4 (6.1%) | 0.861 |
| **Laboratory profiles** |  |  |  |  |
| WBC (×10^9^ /L) | 9.89 (7.65, 12.36) | 10.65 (7.92, 12.80) | 9.44 (6.99, 11.52) | 0.092 |
| RBC (×10^12^ /L) | 4.02 (3.69, 4.42) | 4.05 (3.70, 4.43) | 4.02 (3.69, 4.40) | 0.671 |
| HGB (g/L) | 124.00 (112.00, 135.00) | 124.00 (110.00, 137.00) | 127.00 (115.00, 135.00) | 0.779 |
| PLT(×10^9^ /L) | 167.00 (137.00, 206.00) | 176.00 (140.00, 211.00) | 154.50 (127.00, 192.00) | 0.065 |
| PTINR | 1.12 (1.06, 1.20) | 1.12 (1.03, 1.18) | 1.12 (1.07, 1.21) | 0.104 |
| APTT (s) | 30.20 (28.50, 33.70) | 30.20 (28.40, 34.15) | 30.20 (28.70, 32.60) | 0.738 |
| FIB (g/L) | 3.26 (2.46, 4.31) | 3.45 (2.61, 4.42) | 2.92 (2.33, 3.99) | 0.104 |
| CKMB (ng/ml) | 1.70 (0.70, 6.20) | 1.40 (0.70, 5.00) | 2.80 (0.80, 6.30) | 0.094 |
| CRP (mg/L) | 4.20 (3.30, 5.00) | 4.10 (3.25, 4.95) | 4.35 (3.40, 5.10) | 0.418 |
| NT-proBNP (pg/mL) | 776 (437, 1660) | 742 (420, 1585) | 842 (470, 1720) | 0.326 |
| **Echocardiogram profiles** |  |  |  |  |
| LA (mm) | 35.97 ± 6.58 | 35.51 ± 6.27 | 36.80 ± 7.09 | 0.202 |
| LV (mm) | 45.00 (42.00, 50.00) | 45.00 (43.00, 50.00) | 45.00 (40.00, 50.00) | 0.705 |
| RA (mm) | 44.12 ± 6.75 | 43.24 ± 6.45 | 45.73 ± 7.02 | 0.016 |
| RV (mm) | 23.00 (20.00, 24.00) | 23.00 (20.00, 24.00) | 22.00 (20.00, 24.00) | 0.770 |
| LVEF | 0.60 (0.58, 0.65) | 0.60 (0.58, 0.65) | 0.60 (0.58, 0.65) | 0.753 |
| **Procedure characteristics** |  |  |  |  |
| Root replacement (%) | 76 (41.1%) | 44 (37.0%) | 32 (48.5%) | 0.127 |
| Surgerytime (min) | 480.81 ± 95.81 | 473.74 ± 98.45 | 493.56 ± 90.17 | 0.178 |
| Cardiopulmonary bypass time (min) | 219.00 (189.00, 253.00) | 215.00 (181.50, 249.50) | 224.00 (201.00, 261.00) | 0.075 |
| Aortic cross-clamp time (min) | 134.00 (112.00, 156.00) | 130.00 (108.00, 155.50) | 140.50 (124.00, 163.00) | 0.011 |
| Circulatory arrest time (min) | 22.00 (18.00, 29.00) | 23.00 (19.00, 28.50) | 21.00 (18.00, 30.00) | 0.439 |
| **Perioperative outcomes** |  |  |  |  |
| Operative mortality (%) | 14 (7.57%) | 8 (6.72%) | 6 (9.09%) | 0.572 |

| Non-Cabrol shunt group (n=55) | | | | |
| --- | --- | --- | --- | --- |
|  | Total (55) | Non-POAF group (25) | POAF group(30) | P value |
| **Demographic profiles** |  |  |  |  |
| Female gender (%) | 24 (43.6%) | 9 (36.0%) | 15 (50.0%) | 0.297 |
| Age (years) | 54.00 (41.00, 65.00) | 49.00 (33.00, 60.00) | 57.00 (49.00, 67.00) | 0.035 |
| Weight (kg) | 70.00 (60.00, 80.00) | 68.00 (60.00, 80.00) | 71.00 (62.00, 83.00) | 0.678 |
| **Clinical history and risk factors** |  |  |  |  |
| Smoking history (%) | 19 (34.6%) | 10 (40.0%) | 9 (30.0%) | 0.437 |
| Drinking history (%) | 20 (36.4%) | 9 (36.0%) | 11 (36.7%) | 0.959 |
| Hypertension (%) | 40 (72.7%) | 15 (60.0%) | 25 (83.3%) | 0.053 |
| Diabetes (%) | 2 (3.6%) | 2 (8.0%) | 0 | 0.115 |
| preACS (%) | 10 (18.2%) | 2 (8.0%) | 8 (26.7%) | 0.074 |
| COPD (%) | 3 (5.5%) | 1 (4.0%) | 2 (6.7%) | 0.665 |
| **Laboratory profiles** |  |  |  |  |
| WBC (×10^9^ /L) | 9.79 (7.86, 12.07) | 9.79 (7.99, 12.51) | 9.79 (7.73, 10.88) | 0.408 |
| RBC (×10^12^ /L) | 3.92 (3.36, 4.32) | 3.98 (3.54, 4.34) | 3.79 (3.32, 4.30) | 0.319 |
| HGB (g/L) | 121.00 (102.50, 135.00) | 122.00 (109.00, 138.00) | 119.00 (100.00, 132.00) | 0.398 |
| PLT(×10^9^ /L) | 170.00 (135.00, 211.50) | 175.00 (138.00, 213.00) | 155.50 (131.00, 210.00) | 0.697 |
| PTINR | 1.09 (1.04, 1.17) | 1.09 (1.00, 1.16) | 1.10 (1.00, 1.18) | 0.343 |
| APTT (s) | 31.30 (28.40, 34.25) | 29.40 (27.60, 33.40) | 32.20 (29.30, 34.60) | 0.233 |
| FIB (g/L) | 3.33 (2.76, 3.96) | 3.62 (2.86, 4.35) | 3.19 (2.74, 3.68) | 0.299 |
| CKMB (ng/ml) | 2.00 (1.00, 6.30) | 1.80 (1.00, 5.00) | 2.25 (1.00, 6.30) | 0.588 |
| CRP (mg/L) | 4.90 (3.45, 5.05) | 4.75 (3.35, 5.00) | 5.00 (3.50, 5.10) | 0.452 |
| NT-proBNP (pg/mL) | 829 (488, 1532) | 785 (455, 1480) | 870 (510, 1588) | 0.389 |
| **Echocardiogram profiles** |  |  |  |  |
| LA (mm) | 37.45 ± 6.02 | 36.78 ± 5.72 | 38.00 ± 6.30 | 0.461 |
| LV (mm) | 47.00 (42.00, 51.50) | 45.00 (40.00, 52.00) | 47.50 (45.00, 51.00) | 0.282 |
| RA (mm) | 44.84 ± 8.47 | 43.28 ± 8.32 | 46.13 ± 8.50 | 0.216 |
| RV (mm) | 23.00 (21.00, 25.00) | 23.00 (21.00, 25.00) | 24.00 (21.00, 25.00) | 0.546 |
| LVEF | 0.63 (0.59, 0.65) | 0.64 (0.59, 0.65) | 0.62 (0.56, 0.65) | 0.569 |
| **Procedure characteristics** |  |  |  |  |
| Root replacement (%) | 16 (29.1%) | 9 (36.0%) | 7 (23.3%) | 0.303 |
| Surgerytime (min) | 467.18 ± 85.30 | 474.00 ± 70.19 | 461.50 ± 96.95 | 0.593 |
| Cardiopulmonary bypass time (min) | 206.00 (189.00, 238.50) | 211.00 (182.00, 270.00) | 204.00 (190.00, 228.00) | 0.953 |
| Aortic cross-clamp time (min) | 146.00 (132.00, 162.50) | 138.00 (128.00, 190.00) | 146.50 (133.00, 161.00) | 0.761 |
| Circulatory arrest time (min) | 39.00 (36.00, 42.00) | 38.00 (36.00, 41.00) | 40.00 (36.00, 46.00) | 0.219 |
| **Perioperative outcomes** |  |  |  |  |
| Operative mortality (%) | 4 (7.27%) | 2 (8.00%) | 2 (6.67%) | 1.000 |
